# Judgements of Social Dominance From Faces and Related Variables

**DOI:** 10.3389/fpsyg.2022.873147

**Published:** 2022-04-29

**Authors:** Josefa N. S. Pandeirada, Mariana Madeira, Natália Lisandra Fernandes, Patrícia Marinho, Marco Vasconcelos

**Affiliations:** ^1^William James Center for Research, Department of Education and Psychology, University of Aveiro, Aveiro, Portugal; ^2^Social Services of University of Aveiro, University of Aveiro, Aveiro, Portugal

**Keywords:** social dominance, face evaluation, ratings, social inferences, individual variables, online and laboratory data collection

## Introduction

Assessing someone's dominance from facial cues occurs in a relatively automatic fashion. It allows us to infer power hierarchies as well as the individual's intentions and ability to inflict physical damage (e.g., Todorov et al., 2008). Consistently, such assessment seems to rely mostly on cues of physical strength (e.g., facial masculinity). The adaptive importance of inferring dominance from others is well recognized in the general realm of evolutionary psychology (Oosterhof and Todorov, 2008). Dominance has also been recognized as an important dimension of social and interpersonal perception (e.g., Wiggins, 1979). In fact, Oosterhof and Todorov (2008) have shown that dominance, alongside the evaluation of valence/trustworthiness, is predictive of important social judgments. Furthermore, using the same analytical procedures, Jones et al. (2021), have recently replicated their main conclusion across world regions.

Although the dimension of dominance has been described in multiple contexts and different ways (e.g., Maner, 2017), two distinct (yet not independent) forms have been more closely related to the evaluation of faces: physical and social dominance (e.g., Doll et al., 2014). We note, though, that studies do not always specify which of these forms is being measured (e.g., Oosterhof and Todorov, 2008), casting doubts on what precisely is evaluated by participants.

Physical dominance relates mostly to the use of physical force and intimidation as a means to influence the behavior of others (e.g., Puts et al., 2006, 2007). Assessments of this dimension correlate positively with facial features of masculinity (e.g., Perrett et al., 1998), with measures of actual strength (Fink et al., 2007), and with other facial characteristics signaling physical strength (e.g., Oosterhof and Todorov, 2008; Windhager et al., 2011). Once made, inferences of dominance have real-life impacts. For instance, Flowe (2012) reported that faces perceived as more (vs. less) dominant were rated as having a more criminal appearance, although other variables may mediate such a relation (e.g., the sex of the face and of the rater). These inferences also impact self- and others-perceptions of dominance. Mileva et al. (2014) found that males with higher facial-width-to-height ratio (commonly associated with aggressive behavior) are rated as more dominant by themselves and by others. Furthermore, Doll et al. (2014) found that self-perceptions of fighting ability, but not of leadership ability, agreed with others-perceptions of physical dominance (although see Watkins, Fraccaro et al., 2010).

Inferences of social dominance, on the other hand, seem to rely on cues of social status, including income, education, and social position (Torrance et al., 2014). Some physical features, namely height and facial cues of height (e.g., elongated faces and narrower jaws) relate closely with ratings of social dominance (Windhager et al., 2011). Indeed, taller individuals are preferred as leaders (Murray and Schmitz, 2011) and tend to have higher academic and career success, as well as higher income (e.g., Judge and Cable, 2004). Overall, people with more socially-dominant faces reach a higher social status more easily than people with less dominant faces (e.g., Muller and Mazur, 1997).

The influence of these two forms of dominance in the context of mating can be derived from evolutionary models. Because more dominant males (with more masculine traits) have a greater ability to acquire resources, to provide protection, and have higher quality genes, they usually outcompete their same-sex rivals (Puts et al., 2006; see also Kordsmeyer et al., 2018). Consistently with this functional account, males (but not females) exhibiting cues of social dominance appear to capture more attention of observers (Maner et al., 2008). Other cues of social dominance, including signs of wealth, have also been shown to play a role in the context of mating (e.g., von Rueden and Jaeggi, 2016).

Research has also shown that, even after a mere glimpse, faces alone convey important and reliable dominance information (Rule et al., 2012). For example, dominance ratings made from facial photographs and provided by unfamiliar people are consistent with those made by acquainted people (i.e., those with direct social interaction with the person), as well as with those performed by the individual him/herself (Doll et al., 2014). Furthermore, children as young as three also make dominance inferences from faces that are highly consistent with those provided by adults, suggesting that this ability emerges early in life and requires low levels of social experience (Cogsdill et al., 2014).

As briefly reviewed, inferences of dominance are important in several ways and can be reliably obtained from faces of people. This study presents ratings of social dominance for a large set of stimuli from a European Portuguese sample. We opted to use faces of young-adults with an emotional-neutral expression while recognizing that different emotional expressions impact dominance ratings in various ways (Zebrowitz, 2017). We collected our data from an online and a laboratory sample also contributing to the ongoing debate about the reliability of data collected online. Furthermore, dominance assessments were collected together with other variables known to relate or influence the assessment of dominance, such as the participants' age, sex and height, self- and others-perceived dominance, as well as self-attractiveness. Studies exploring how dominance judgments may change as the individual ages throughout adulthood are still scarce and the existing results are mixed. Some studies report that facial dominance is evaluated similarly by different age groups (Cortes et al., 2019), whereas others show that older adults (vs. younger adults) judge faces as generally more dominant (Kiiski et al., 2016). Given that our participants were from a wide age range (particularly those from the online sample), our data should contribute to clarify this issue.

Regarding sex, dominance tends to be judged similarly by males and females (e.g., Mignault and Chaudhuri, 2003). However, the sex of the face being rated affords different inferences: in general, male faces tend to be rated as more dominant than female faces (e.g., Mignault and Chaudhuri, 2003) even though the type of dominance under evaluation also seems to play a role (e.g., Watkins, Jones et al., 2010). Our data will potentially help clarify these questions. The individuals' height also seems to influence the extent to which he/she is sensitive to other peoples' cues of dominance (e.g., Watkins, Jones et al., 2010; Watkins et al., 2012).

Finally, it has been shown that exposure to other individuals varying in their dominance and attractiveness impact the individuals' own assessments on these two dimensions (Gutierres et al., 1999). Even though this was not directly manipulated, relations among them may be explored in our dataset. Besides providing an evaluation of self- and others-perceived dominance, participants also indicated their fighting and leadership abilities, and their socioeconomic status as proxy measures of dominance (e.g., Doll et al., 2014).

## Method

### Participants

#### Online Sample

A total of 486^1^ participants (age range: 18–69; *M*_age_ = 35.80, *SD* = 12.65) filled the entire questionnaire until completion. Data from other 50 participants were excluded (for not being European Portuguese participants, *n* = 41; and due to randomization errors in the questionnaire, *n* = 9).

#### Laboratory Sample

Data were obtained from 138 participants (age range: 18–35; *M*_age_ = 21.38, *SD* = 3.44). Ten other participants were not included due to randomization errors in the program (*n* = 2) or for not being European Portuguese participants (*n* = 8).

Table 1 presents a complete characterization of both samples regarding sex, age group, and years of education.

**Table 1:** Characterization of the sample regarding sex, age group, and years of education.

	**Online sample**		**Laboratory sample**	
	***N***	**Percentage**	***N***	**Percentage**
**Sex**				
Female	318	65.4	110	79.7
Male	168	34.6	28	20.3
**Age group**				
Young adults (18–29 years)	193	39.7	131	94.9
Middle-aged adults (30–49 years)	201	41.4	7	5.1
Older adults (≥50 years)	92	18.9	0	0
Years of Education^*^				
≤12 years of education	56	11.5	30	21.7
>12 years of education	429	88.5	108	78.3

* *One participant from the online sample did not provide information on his/her years of education*.

### Material

Using the same procedure used by Pandeirada et al. (2020), two of the authors selected faces from the following databases: (1) Karolinska Directed Emotional Faces (KDEF; Goeleven et al., 2008); (2) Warsaw Set of Emotional Facial Expression Pictures (WSEFEP; Olszanowski et al., 2014); (3) Radboud Facial Database (RaFD; Langner et al., 2010); (4) FACES Database (Ebner et al., 2010); and (5) Amsterdam Dynamic Facial Expression Set (ADFES; van der Schalk et al., 2011). This selection resulted in a set of 231 frontal-view pictures of colored young adult facial photographs (105 females and 126 males) with similar characteristics to those of the Portuguese population, displaying direct eye gaze and a neutral facial expression, and that were obtained under controlled conditions. Some of the photos were edited to obtain a homogeneous set of stimuli across all databases (as in Pandeirada et al., 2020). Required written consents were obtained from the respective authors and/or laboratories.

Each participant rated 50 faces which were pseudo-randomly selected from the total set of stimuli while ensuring that the same number of female (*n* = 25) and male faces (*n* = 25) was presented in each questionnaire, and that the number of faces selected from a given database was proportionally similar across databases (see the distribution of stimuli by database in the datasets available on https://osf.io/pm2tc/?view_only=2aa89739166742e1b4344b93c5ccd809).

### Procedure

Data were collected online and in the laboratory from March to September of 2015 using a questionnaire programmed in LimeSurvey. The online data were obtained through e-mail dissemination of the task (e.g., universities, professional schools, and companies). Participants recruited at the Universities of Aveiro, Minho, and Coimbra provided the laboratory data. All participants were required to be at least 18 years and of Portuguese nationality.

We used that same questionnaire with both samples. The questionnaire started with a proper consent form and then the program moved on to collect information about the participants' sex, age, height, nationality, marital status, years of education, and whether they were a college student. The dominance rating procedure then followed. Participants were informed that they would see faces presented sequentially and should rate how dominant each face was to them using a 7-point rating scale, where 1 corresponded to “not dominant at all” and 7 to “very dominant”; responses were given by selecting the radio button that corresponded to their choice. Dominance was defined as “the disposition and ability of an individual to be influential, respected, and often a leader.” Participants were given unlimited time to respond to each face but were instructed to respond quickly and to rely on their “gut instinct.” Participants were also told that their responses represented their personal view and that there were no correct or incorrect evaluations.

Only one face was presented per trial. Each appeared at the center of the screen on a white background, in a different order for each participant; the response scale was presented below it. Each picture was preceded by a 1000-ms fixation cross and followed by a 500-ms blank screen.

After rating the faces, participants rated their own dominance and how dominant they thought other people would rate them; the same 7-point rating scale was used. Then they provided information on three characteristics closely related to dominance (Doll et al., 2014): (1) fighting ability: if entering in a physical fight, what percentage of individuals of their own sex and age they could defeat (0–100% rating scale, with increments of 10%); (2) leadership ability: how good a leader they were (1–7 rating scale); and, (3) self-attractiveness: how attractive they were (1–7 rating scale). Finally, they were asked to indicate their socioeconomic status by choosing one of five options, ranging from very low to very high socioeconomic status.^2^ The questionnaire ended with a final appreciation message.

## Results

An average of 103.2 (*SD* = 15.59; range: 56–148) and 29.9 (*SD* = 6.06; range: 13–48) ratings per face were obtained from the online and laboratory samples, respectively. The variation in the number of ratings per face resulted from the pseudo-random selection of the 50 faces to present in each questionnaire. Detailed rating information is provided in the data files made available through OSF (https://osf.io/pm2tc/?view_only=2aa89739166742e1b4344b93c5ccd809). File “Dominance_SubjectDatafile_OSF.xlsx” reports the information collected from each participant (“Participant Data” worksheet); the “Read Me First” worksheet describes the presented data. File “Dominance_Item Datafile_OSF.xlsx” contains the information presented by item and includes, for each face, the number of collected evaluations and the average rating (and corresponding SD), separately for each sample. Data are also presented separately according to some individual variables as listed below.^3^ The item-data file includes the following worksheets:

1. Read Me First: Describes the information presented in each worksheet. At the bottom of this tab, a table describes the number of faces presented in each questionnaire from each database;
2. Overall Data: Presents the overall dominance ratings;
3. Sex: Presents dominance ratings depending on the sex of the participant;
4. Age Group: Data are provided separately for “young-adults,” middle-aged” and “older adults,” following the criteria from McLellan and McKelvie (1993);
5. Years of Education: Ratings are provided broken down by the participants' years of education (≤12 or >12);
6. Self and Others' Evaluation: Ratings are provided separately according to participants' self and others evaluations: low (ratings of 1–2), average (ratings of 3–5), and high dominance (ratings of 6–7).

Table 2 reports the mean number of ratings and mean dominance values per face, and separately for the female and male faces. These data are also presented broken down by sex, age group, and number of years of education, from the online and the laboratory samples.

**Table 2:** Mean number of responses and mean dominance ratings, and corresponding standard deviations (SD), for all faces and separately for the female and male faces.

	**All faces**				**Female faces**				**Male faces**			
	**Mean number of responses (SD)**		**Mean rating (SD)**		**Mean number of responses (SD)**		**Mean rating (SD)**		**Mean number of responses (SD)**		**Mean rating (SD)**	
**Raters**	**Online**	**Lab**	**Online**	**Lab**	**Online**	**Lab**	**Online**	**Lab**	**Online**	**Lab**	**Online**	**Lab**
Full sample	103.15 (15.59)	29.87 (6.06)	3.68 (0.61)	3.59 (0.76)	112.32 (16.27)	32.86 (5.58)	3.76 (0.55)	3.79 (0.66)	95.51 (9.87)	27.38 (5.29)	3.61 (0.66)	3.41 (0.80)
**Sex**												
Female	67.45 (11.71)	23.81 (5.17)	3.69 (0.63)	3.53 (0.82)	73.49 (12.21)	26.19 (4.99)	3.78 (0.56)	3.80 (0.71)	62.43 (8.50)	21.83 (4.44)	3.62 (0.67)	3.30 (0.84)
Male	35.70 (6.65)	7.11 (1.64)	3.65 (0.64)	3.81 (0.74)	38.84 (7.08)	7.38 (1.60)	3.72 (0.59)	3.71 (0.73)	33.08 (4.94)	6.83 (1.65)	3.58 (0.68)	3.92 (0.75)
**Age group**												
Young adults (18–29 years)	40.90 (7.72)	28.35 (5.88)	3.59 (0.67)	3.59 (0.78)	44.58 (8.01)	31.19 (5.44)	3.66 (0.59)	3.81 (0.68)	37.83 (5.94)	25.99 (5.15)	3.53 (0.72)	3.41 (0.81)
Middle-aged adults (30–49 years)	42.71 (7.88)	–	3.69 (0.64)	–	46.49 (8.47)	–	3.79 (0.57)	–	39.56 (5.71)	–	3.61 (0.68)	–
Older adults (≥50 years)	19.54 (4.50)	–	3.84 (0.64)	–	21.26 (4.58)	–	3.91 (0.63)	–	18.11 (3.91)	–	3.78 (0.64)	–
**Years of education**												
≤12 years of education	11.94 (3.29)	7.40 (2.01)	3.78 (0.72)	3.45 (1.01)	12.98 (3.27)	7.83 (2.09)	3.89 (0.72)	3.73 (0.90)	11.08 (3.05)	6.98 (1.85)	3.69 (0.72)	3.18 (1.04)
>12 years of education	91.07 (14.24)	23.38 (5.02)	3.67 (0.62)	3.62 (0.75)	99.20 (14.90)	25.71 (4.47)	3.75 (0.55)	3.80 (0.65)	84.29 (9.30)	21.43 (4.62)	3.60 (0.66)	3.46 (0.80)

*Data are also presented broken down by sex, age group, and years of education. These data are also broken down by sample (online and laboratory samples)*.

*Data on some variables are not presented as the overall mean number of ratings was lower than 5 (see the Datafiles for more details)*.

## Discussion

A large body of literature suggests that inferring dominance from faces is a highly adaptative process that occurs from very early in our development. Its impact on behavior in a myriad of domains makes it an important topic of research that requires properly validated stimuli. Here, we report dominance ratings for a large set of faces obtained from the Portuguese population. To our best knowledge, only one previous study has provided similar norming data from our population but using only the Productive Aging Laboratory Face Database and focusing exclusively on young adult respondents (Ramos et al., 2016).

The ratings provided here should be useful for researchers from various areas wishing to choose stimuli that vary on social dominance, control them on this dimension, or simply consider it when analyzing their data. Given that some studies support a relatively widespread cross-cultural agreement on several trait inferences from faces (e.g., Sutherland et al., 2018), we expect our data to be useful for researchers from other countries and cultures. Favoring this argument, an exploratory inspection of the agreement between our ratings and those obtained in the Netherlands by Jaeger (2020) for faces from the RaFD revealed an excellent level of agreement (Intra Class Correlation Coefficient [ICC] of 0.93 and 0.90, for young adults' data collected in the laboratory and online, respectively). Nonetheless, compared to other inferences, the evaluation of dominance seems to be more flexible and susceptible to contextual factors (e.g., Sutherland et al., 2020). This variability might be related to the somewhat loose specification of dominance in several studies. This is a crucial aspect as the interplay between several variables (e.g., sex of the rater and of the face) and inferences of dominance seem to depend on such definition (Watkins, Jones et al., 2010). We provided a clear instruction for evaluating social dominance, a facet that is less prone to cultural differences (e.g., Sutherland et al., 2018), ensuring researchers will know what they will be manipulating and/or controlling.

Besides making available normative information for a large set of stimuli, we also provide data on other variables that have been, one way or another, shown to influence or relate to evaluations of dominance (e.g., Watkins, Fraccaro et al., 2010). Our data can be used to enlighten some of the ongoing debates involving this inference, such as the effect of sex (of the rater and of the face) or the influence of age throughout adulthood on dominance assessments. Regarding the ongoing examination of the reliability of online data collection, our results revealed an excellent agreement between the data collected in both settings (ICC = 0.92). Hence, in line with previous studies (e.g., Walter et al., 2019), we provide another piece of evidence favoring online data collection procedures.

Interesting interplays have been reported between the evaluations of dominance and those of other dimensions that strongly impact social interactions in several ways. For example, a combination of dominance and valence/trustworthiness judgments seems to underlie the structure of facial impressions, although not totally independent; trustworthy faces (i.e., more feminine traits) are usually regarded as non-dominant and untrustworthy faces as dominant (Todorov, 2017). Furthermore, the way the different components interact to form trait impressions might differ depending on the region of the world being considered and on how data are analyzed (Jones et al., 2021).

Attractiveness and dominance cues also seem to interact in mating contexts. For example, increased dominance evaluations are often associated with perceptions of higher attractiveness in males, irrespective of the rater's sex (e.g., Keating, 1985). In contrast, traits that raise dominance and attractiveness ratings in male faces reduce the perceived attractiveness of females faces (e.g., Oh et al., 2019). Additionally, the extent to which these two relate and/or interact seems to depend both on the sex of the rater and of the to-be-rated face (e.g., Doll et al., 2014).

Importantly, our research team has collected ratings for the current set of faces on the dimensions of attractiveness (Pandeirada et al., 2020) and trustworthiness (Pandeirada et al., 2022). In both instances, we provide a brief overview of the impact that these inferences have on social interactions. As in the present case, information on other variables that are closely related to each dimension is also reported. Having norming data for the same faces spanning attractiveness, trustworthiness and dominance allows for a more careful and integrated stimuli-selection strategy, more detailed data analyses, and opens new research opportunities in several domains.

In sum, these normative data, hereby made available to the scientific community, provide a valuable tool to those interested in studying this dimension or including it in their studies. We also expect these data will help to clarify and expand our knowledge on some of the relations reported in the literature between dominance and other variables.

## Data Availability Statement

The datasets presented in this study can be found in online repositories. The names of the repository/repositories and accession number(s) can be found below: https://osf.io/pm2tc/?view_only=2aa89739166742e1b4344b93c5ccd809.

## Ethics Statement

Ethical review and approval was not required for the study on human participants in accordance with the local legislation and institutional requirements in place at the time the study was conducted. The patients/participants provided their written informed consent to participate in this study. All procedures were in accordance with the 1964 Helsinki declaration and its later amendments, in particular with the following aspects: participation in the project added no risk or burden to participants, the benefits of the project clearly outlined its potential risks, written informed consent was obtained from all participants, participation was voluntary, and data were managed and are presented in a manner that ensures the participants' anonymity.

## Author Contributions

JP and NF developed the idea and procedures to collect the data. NF managed the data collection process. MM, JP, and PM contributed to the writing of the Introduction. MM and JP provided preliminary analysis and worked on the Discussion. All authors contributed to the writing, discussion, commenting of the manuscript, and approved the final version of this manuscript.

## Funding

The Portuguese Science Foundation provided support to JNSP (Research and Investigator Grant, ref. PTDC/MHC-PCN/5274/2012, IF/00058/2012/CP0172/CT0002, and CEECIND/01914/2017), to NF and PM (Research Grant PTDC/MHC-PCN/5274/2012). MM was supported by a research grant awarded by the University of Aveiro. Support was also attained through the WJCR (UID/04810/2020).

## Conflict of Interest

The authors declare that the research was conducted in the absence of any commercial or financial relationships that could be construed as a potential conflict of interest.

## Publisher's Note

All claims expressed in this article are solely those of the authors and do not necessarily represent those of their affiliated organizations, or those of the publisher, the editors and the reviewers. Any product that may be evaluated in this article, or claim that may be made by its manufacturer, is not guaranteed or endorsed by the publisher.

## Footnotes

^1^Due to a programming error, about 52% of the sample responded to a questionnaire that did not display faces from the ADFES database.

^2^Female participants also provided information about their menstrual cycle (average duration, date of last menstrual cycle, and use of oral contraceptives).

^3^When the average number of ratings on a given variable condition was lower than five, no data are provided for that condition. Information for a given face is not shown when the number of ratings for that face was less than five. These occurred mostly for the laboratory data, due to the smaller sample size and higher homogeneity (all details are provided in the data file).
